# An Evaluation of Flavored Photostimulable Phosphor (PSP) Barrier in Bitewing Radiography: A Randomized Crossover Study

**DOI:** 10.1002/cre2.70329

**Published:** 2026-03-26

**Authors:** Shwetha Hegde, Scott Cameron Dickie, Thirimadura Ruvin Mendis, Mathew Yu, Shanika Nanayakkara, Amelita Simpson, Wendy Currie

**Affiliations:** ^1^ Dentomaxillofacial Radiology, Sydney Dental School University of Sydney Sydney Australia; ^2^ Sydney Dental School University of Sydney Sydney Australia; ^3^ Sydney Dental School, Institute of Dental Research, Westmead Centre for Oral Health University of Sydney Sydney Australia

**Keywords:** barrier sleeves, bitewing radiography, patient acceptability, photostimulable phosphor plates, randomized crossover trial

## Abstract

**Objectives:**

To assess whether flavoured PSP barrier sleeves enhance patient comfort and sensory perception during intraoral radiography compared to non‐flavoured sleeves.

**Material and Methods:**

In a double‐blind, randomized, crossover study, 74 dental students underwent two molar bitewing placements in random order, with a 15‐min washout period. Participants rated overall comfort, gagging, oral irritation, taste, aftertaste, scent, feel, and overall procedure experience. Ordinal outcomes used the Wilcoxon signed‐rank test, and binary outcomes used McNemar's test. Sequence and carryover effects were examined with the Mann–Whitney *U* test. Significance was set at *p* < 0.05.

**Results:**

Flavored sleeves scored higher for chemosensory experience (*p* < 0.001; Cohen's *r* > 0.5). Order effects were noted for overall comfort, taste, aftertaste, and scent (*p* < 0.001; moderate to large effect size). The carryover effect persisted for taste, scent, and aftertaste (*p* < 0.01). Preference for flavored sleeve was 58.7% and strongly linked to treatment order (*χ* ^2^ = 11.70, *p* < 0.01).

**Conclusions:**

Flavored PSP barrier sleeves enhance chemosensory experience and perceived acceptability of bitewing radiography, with a limited impact on procedural discomfort and gagging. Their use may improve patient cooperation and experience in clinical settings.

**Clinical Relevance:**

Bitewing radiography is commonly used, but it can be uncomfortable due to the film positioning, patient cooperation, and the taste and feel of the barrier sleeves used with photostimulable phosphor plates. In this randomized crossover study, mint‐flavored barrier sleeves improved chemosensory experience and patient acceptability. These findings present a low‐cost option to enhance the intraoral radiography experience, particularly for first‐time, anxious, or pediatric patients.

## 1 Introduction

1.1

Bitewing radiographs are routinely used in dentistry for diagnosis, treatment planning, and follow‐up and producing high‐quality radiographs is essential for accurate diagnosis (Rozylo‐Kalinowska 2020). With the widespread adoption of digital radiography, clinicians can choose between solid‐state detectors (SSDs), such as charge‐coupled devices and complementary metal‐oxide‐semiconductors, or photostimulable phosphor (PSP) receptors (Dean 2015). PSP receptors offer several advantages over SSD, including smaller size, a thinner profile, increased flexibility, ease of receptor placement in the patient's mouth and improved image quality (Gonçalves et al. 2009). However, intraoral radiography, particularly molar bitewings, can be uncomfortable for the patient. Factors such as the size and thickness of the receptor, positioning, physical discomfort due to the receptor impinging on the soft tissues in the lingual sulcus, and gagging can negatively impact the patient's experience (Jørgensen and Wenzel 2012; Zhang et al. 2019).

Beyond mechanical factors contributing to discomfort, patient experience during intraoral radiography is shaped by the chemosensory context (taste and smell), attention, and anxiety. Studies have shown that pleasant olfactory and taste cues in dental settings can reduce state anxiety and improve perceived experience, supporting targeted sensory adjustments to routine procedures (Cai et al. 2021; Arslan et al. 2020). In bitewing placement specifically, soft tissue impingement and the perceived bulk of the positioning aids and receptors may affect tolerance. They may result in poor quality non‐diagnostic images and unnecessary radiation exposure to the patient (Zhang et al. 2019).

PSP receptors must be enclosed in single‐use barrier sleeves for infection control, and anecdotal reports suggest that the plastic‐like taste and aftertaste affect the patient experience. Efforts to improve patient comfort during intraoral radiography have primarily focused on technique modifications, positioning aids and distraction methods (Madan et al. 2015; Dailey and Brooks 2019). However, sensory discomforts such as an unpleasant taste, aftertaste, and a plastic‐like feel of barrier materials remain unaddressed. Although flavoring agents are commonly used in dentistry, such as fluoride gels, prophy pastes, and impression materials, to improve patient acceptance and experience, the application of such modifications to PSP sleeves has not been explored in the literature (Cugati 2012; Eriwati et al. 2021). There is a knowledge gap regarding whether flavored enhanced barrier sleeves can reduce patient discomfort and improve the patient experience during intraoral imaging.

This study evaluated whether flavored PSP barrier sleeves improve patient comfort and sensory perception during intraoral radiography compared to non‐flavored sleeves. The primary hypothesis was that flavored sleeves would be rated more favorably by participants than non‐flavored ones. This study aimed to address the following objectives:

1. To determine whether adding flavoring to PSP barrier sleeves improves the patient's overall experience during intraoral radiography.
2. To determine the effects of flavoring on PSP barrier sleeves on participants’ self‐reported outcomes, including comfort, the likelihood of gagging, scent, initial taste, aftertaste, and oral irritation.

## 2 Methods

This study was conducted as a single‐center, randomized, double‐blind, crossover study. A cross‐over design is a study design in which each participant receives all the interventions in separate time periods, in a randomized order, with a washout period in between, so each participant serves as their own control (Lim and In 2021). It received ethics approval from the University of Sydney Ethics Committee (approval number 2022/784). The sample size was estimated at 74 participants based on a previous study (Bailey et al. 2018). The calculation was based on 80% statistical power and a 0.05 significance level, with a 1:1 ratio in the two groups, assuming that 30% more participants prefer flavored PSP than those without flavoring.

### 2.1 Participant Recruitment and Eligibility

Convenience sampling was undertaken among students enrolled in the Doctor of Dental Medicine (DMD) and Bachelor of Oral Health (BOH) programs. Enrollment in the study was voluntary, and student recruitment was conducted through Canvas, a learning management system used at Sydney Dental School. Interested participants completed an online consent form hosted on the Qualtrics platform (University of Sydney). The consent form included questions to identify participant eligibility for the study (Supporting Information File 1). All participants first completed a brief screening questionnaire (hosted on Qualtrics) to document any history of gagging, allergies to study materials, and prior experience with intraoral radiography, and to identify exclusion criteria. This questionnaire also included a question about the participants’ flavor preference (mint, strawberry, bubble gum, chocolate, vanilla, and unflavored) on a ranking scale from 1 (most preferred) to 6 (least preferred) (Supporting Information File 2).

### 2.2 Participants Were Excluded If They Were

1. Recovering from recent oral surgery
2. Had known allergies to the materials used in the study
3. Currently positive with COVID‐19
4. Under the age of 18
5. Smokers
6. Pregnant or potentially pregnant

In appreciation of their time and participation, each participant was given a plastic typodont tooth (One Dental Pty Ltd.).

### 2.3 Study Procedure

**Figure 1:**
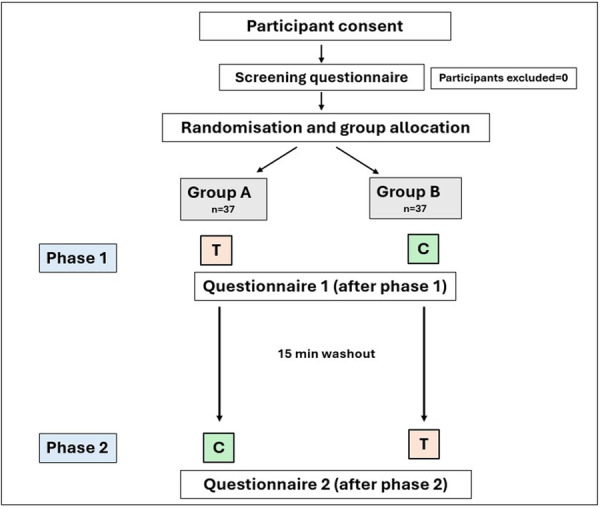
Study design showing group allocation of participants. (T = treatment, flavored barrier sleeve; C = control, non‐flavored barrier sleeve.)

The study procedure, illustrated in Figure 1, involved placing a size 2 PSP film in a disposable, single‐use barrier sleeve with a loop‐type cardboard bitewing tab, then placing it in the mouth for a molar bitewing and leaving it in place for 30 s. Molar bitewings with disposable tabs were used, as this is a common clinical scenario, reducing the need for sterile instrumentation and simplifying setup and infection control logistics for a crossover design. In addition, the rigid positioning devices, whose greater (perceived) bulk can confound chemosensory effects by introducing additional mechanical discomfort. To standardize the flavor intervention, barrier sleeves were prepared by a non‐assessing investigator in a clean, dedicated area as follows: (1) 2 pumps of mint breath spray per side delivered from approximately 5 cm distance to the outer surfaces of the PSP sleeve, (2) the sleeves were air dried on sterile surfaces for 10 min, and (3) the prepared sleeves were used in the experiment immediately after drying. The control sleeves were handled identically without the use of spray. All sleeves were visually indistinguishable. The spray used in this study was a commercially available mint breath spray (12 mL) with labeled ingredients including ethanol, water, glycerin, PEG‐40 hydrogenated castor oil, flavor/peppermint oil, methyl salicylate, sodium saccharin, menthol, cetylypyridinium chloride, and permitted colorants (CI 18965, CI 42090); exact composition was documented from the manufacturer's public ingredient disclosure at the time of study setup. The mint flavor was selected because the mint breath spray was commercially available over the counter and labeled for intraoral use. In addition, mint is the most commonly encountered flavor in daily use oral care products, making it a familiar and broadly acceptable flavor. The chosen spray was also fast evaporating and colorless, leaving minimal residue on the PSP barrier sleeves.

### 2.4 Randomization and Blinding

Participants were assigned a participant ID and randomly assigned to one of the two groups, either Group A (flavored PSP first) or Group B (non‐flavored PSP first), using an online randomizer (www.randomiser.org). Randomization was conducted without stratification, and allocation concealment was ensured by storing the randomization list in a secure Excel file managed by an investigator (S.C.D.) in the research team who was not involved in data collection or analysis. This investigator maintained the randomization list linking participants’ IDs to their respective treatment phases. This investigator (S.C.D.) also prepared the PSP plates and assigned participants to their respective groups according to the randomization list. The participants and all other members of the research team were blinded to the group allocations. The flavored and non‐flavored PSP barrier sleeves were indistinguishable in appearance to ensure effective blinding.

### 2.5 Experimental Protocol

During the experiment, the participants were seated upright in a dental operator's chair. The PSP film with the cardboard bitewing tab was placed in the mouth for a molar bitewing and left for 30 s (Phase 1). This procedure was timed, and the PSP was removed after 30 s; no radiograph was taken. After a timed 15‐min washout period, the groups were crossed over, and the same procedure was repeated by placing the film for a bitewing on the other side of the mouth (Phase 2). Participants in Group A received a PSP film with a barrier sleeve with flavoring first, while Group B received a PSP film with an unflavored barrier sleeve first.

### 2.6 Questionnaire Data Collection

At the end of each phase (treatment or control), the participants completed a questionnaire hosted on Qualtrics. The questionnaire was conveniently accessed via a QR code, which participants scanned with their mobile phones. This questionnaire was designed to evaluate various aspects of the participant experience, using a combination of Likert‐scale items and open‐ended text boxes. The following variables were assessed:

1. Overall comfort: the general sense of ease during the procedure, including tolerability of PSP and impact on the participant's overall comfort. Rated on a 5‐point Likert scale (1=very uncomfortable, 5=very comfortable).
2. Gagging: the extent of the gag reflex during the procedure (yes/no). If they answered yes, they were asked to rate the strength of the gag reflex on a 5‐point Likert scale (1= very mild, 5 = very severe).
3. Oral irritation: the presence of oral discomfort or irritation (yes/no). Those who answered “yes” were asked to rate its severity on a 5‐point Likert scale (1 = very high, 5 = very low).
4. Experience with the scent of PSP: acceptability and pleasantness of the smell of PSP, rated on a 5‐point Likert scale (1 = very uncomfortable, 5 = very comfortable).
5. Experience with the taste of PSP: flavor of the barrier sleeve, whether it enhanced or detracted from the participant's experience, rated on a 5‐point Likert scale (1 = very uncomfortable, 5 = very comfortable).
6. Experience with the aftertaste of PSP: the presence of a lingering taste after the procedure, rated on a 5‐point Likert scale (1 = very uncomfortable, 5 = very comfortable).
7. Experience with the feel of PSP: the tactile sensation of the PSP against the oral tissues, including its texture and fit, rated on a 5‐point Likert scale (1 = very uncomfortable, 5 = very comfortable).
8. Overall procedure: A holistic evaluation of the entire procedure, considering all aspects of the participant's experience, rated on a 5‐point Likert scale (1 = very uncomfortable, 5 = very comfortable).

At the end of Phase 2 of the study, an additional question was included in Questionnaire 2 to assess participants’ preferences for the barrier sleeve (flavored or unflavored) (Supporting Information Files 2 and 3).

### 2.7 Data Analysis

Statistical analyses were conducted using SPSS (version 29, IBM SPSS Inc., Chicago, IL).

Descriptive statistics were used to summarize participant demographic data, prior radiographic experiences, and self‐reported gagging history. Frequencies and percentages were calculated for categorical variables, and medians with interquartile ranges (IQR) were used to describe ordinal data. The participants’ rankings for flavor preference were analyzed using Friedman's test.

Ordinal paired outcomes (comfort, taste, aftertaste, scent, feel, overall procedure) were compared between flavored and non‐flavored sleeves using the Wilcoxon signed‐rank tests and oral irritation using McNemar's test. Effect sizes for Wilcoxon tests were calculated using Cohen's *r* (interpreted as small: *r* = 0.10–0.29; moderate: *r* = 0.30–0.49, and large: *r* ≥ 0.50).

To evaluate order effects, Mann–Whitney *U* tests were used to compare difference scores between Phase 1 and 2 responses. Chi‐square tests were used to compare proportions across the treatment order groups for categorical outcomes. Within‐group comparisons (Phase 1 vs. Phase 2) were also performed separately for Group A (flavored first) and Group B (non‐flavored first) using Wilcoxon signed‐rank tests. Where appropriate, effect sizes for Wilcoxon and Mann–Whitney tests were calculated and interpreted according to conventional methods. A significance level of *p* < 0.05 was used for all tests.

Unblinding was done after data collection and analysis were completed to maintain the integrity of the double‐blind study design. The allocation codes for each participant, which an investigator in the research team securely maintained throughout the study, were accessed only after all the statistical analyses were finalized and verified. Once unblinding was complete, the treatment allocations were matched with the outcomes to facilitate interpretation of the results and to understand the impact of the flavored PSP barrier sleeve on participants’ experiences.

**Figure 2:**
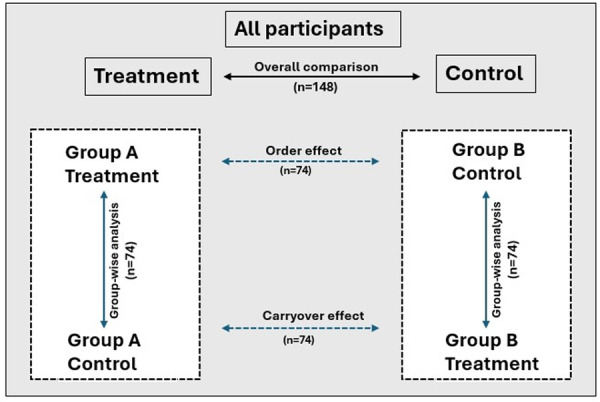
Data analysis plan. Overall comparison between flavored and non‐flavored PSP sleeves across all participant responses. Within‐group comparisons of treatment (flavored) and control (non‐flavored) responses in each group separately. Order effect analyses comparing Phase 1 outcomes between Group A and Group B. Carryover effect was assessed by comparing the Phase 2 outcomes between Group A and Group B.

The data analysis plan is depicted in Figure 2.

## 3 Results

A total of 74 DMD and BOH students participated in this study. The majority of participants (*n* = 69; 93%) had prior experience with intraoral radiographs, and their experience varied. Among participants, 42% (*n* = 31) reported experiencing a gagging problem, with varying severity, while the majority (*n* = 43; 58%) indicated that they did not have a gagging problem. Of the 31 participants who reported a gagging problem, 54.8% (*n* = 17) stated that they had experienced a negative incident related to gagging, and 51.6% (*n* = 16) reported having gagged at the dentist previously, with them experiencing gagging during impression taking, intraoral radiography, and teeth cleaning (Table 1).

**Table 1:** Participant characteristics, prior radiographic experiences and self‐reported history of gagging experience.

Variable	Response	*n* (%)
Prior experience with intraoral radiographs	Yes	69 (93)
	No	5 (7)
Prior radiographic experience rating (*n* = 69)	Extremely good	12 (17.4)
	Somewhat good	26 (37.7)
	Neither good nor bad	26 (37.7)
	Somewhat bad	5 (7.2)
	Extremely bad	0
Gagging problem	Yes	31 (42)
	No	43 (58)
Gagging severity^a^ (*n* = 31)	Normal gagging	2 (6.5)
	Mild gagging	7 (22.6)
	Moderate gagging	14 (45.2)
	Severe gagging	7 (22.6)
	Very severe gagging	1 (3.2)
Negative experience with gagging (*n* = 31)	Yes	17 (54.8)
	No	14 (45.2)
Ever gagged at the dentist? (*n* = 31)	Yes	16 (51.6)
	No	15 (48.4)

*Note:* Shaded cells indicate responses to follow‐up questions that were only asked of participants who answered “yes” (*n* = 31) to the initial gagging question.

a Adapted from the classification of gagging problem (CGP) index (Hearing et al. 2014) 5‐level scale to measure the severity of the patient's gag reflex.

A Friedman test revealed a significant difference in participants’ flavor preferences (*χ* ^2^(5) = 45.81, *p* < 0.001). The mean ranks indicated that mint (*M* = 2.49) and strawberry (*M* = 2.78) were the most preferred options, while the unflavored option was the least preferred (*M* = 4.62) (Table S1).

**Figure 3:**
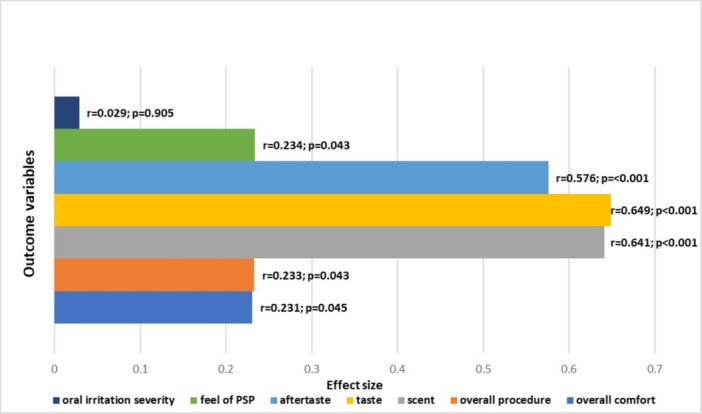
Comparison of effect size and *p* values for different variables in flavored and non‐flavored groups.

1. Comparison of participant responses (flavored vs. non‐flavored sleeves):
  Analysis revealed statistically significant differences in participant responses between the flavored and non‐flavored PSP barrier sleeves. Specifically, participants rated the flavored sleeves significantly higher for taste, aftertaste, scent, overall procedure, feel, and overall comfort, with moderate to large effect sizes (Figure 3). Out of 74 participants, 17 (23%) indicated that they experienced oral irritation from flavored PSP, and 18 (24.3%) experienced oral irritation from non‐flavored PSP. Gagging was reported by five participants during the placement of flavored sleeves and seven during the placement of non‐flavored sleeves. However, McNemar's test indicated no significant differences between the two sleeve types for oral irritation (*p* = 1.000) and gagging (*p* = 0.687). Among the participants who reported oral irritation, there was no statistically significant difference in oral irritation severity ratings (Likert scale) between flavored and non‐flavored PSP sleeves (*Z* = −0.119, *p*‐value = 0.905). Given the small number of participants who responded “yes” to experiencing gagging, the follow‐up Likert scale ratings of severity were not analyzed further. A summary of the significant findings, including effect sizes, is presented in Table S2.
2. Within‐group comparisons:
  Participants’ responses to the treatment and control phases were compared within Group A and Group B using the Wilcoxon test (Table 2). In Group A (participants received flavored sleeves first), significant differences were observed in favor of flavored sleeves across multiple variables. Specifically, taste, scent, aftertaste, and overall procedure showed large effect sizes, indicating higher participant ratings for flavored sleeves. For the feel of PSP, all participants gave identical ratings for both sleeve types, and the differences were non‐significant (*p* = 1.000) with no measurable effect size.
  In Group B (participants received non‐flavored sleeves first), the same pattern was observed. The flavored sleeve (received second) was rated significantly higher for taste, scent, and aftertaste, all with large effect sizes. A moderate effect was observed for feel. However, there were no significant differences in overall comfort, overall procedure, and oral irritation (severity), with negligible effect sizes for overall comfort and overall procedure. For oral irritation severity, although the effect size was moderate, the severity scores did not differ significantly between the two conditions. These within‐group findings support the sensory advantage of the flavored PSP sleeves, regardless of the order in which they were received.
  For oral irritation severity, in both Groups A and B, although the effect sizes were moderate, the median and IQR were both 0 for the full sample, reflecting a strong floor effect. This, along with a non‐significant *p*‐value, suggests no meaningful differences in responses to flavored and non‐flavored sleeves.
3. Order effect analysis:
  Participants who received the flavored sleeve first reported significantly higher scores for overall comfort, taste, aftertaste, and scent, with moderate to large effect sizes. No statistically significant differences were observed for oral irritation severity (*p* = 0.562), the feel of PSP (*p* = 0.275), or the overall procedure (*p* = 0.072) (Table 3). Chi‐square tests conducted for oral irritation experience (categorical variable) revealed no significant difference in oral irritation (*χ* ^2^ = 0.53, *p* = 0.466) between Groups A and B, with no order effects. A statistically significant difference in gagging was observed between groups (*χ* ^2^ = 4.11, *p* = 0.043), indicating a possible order effect, suggesting that participants who received the non‐flavored sleeve first were more likely to experience gagging during Phase 1.
4. Carryover effect analysis:
  To examine potential carryover effects, responses from Phase 2 were compared between Group A and Group B. Mann–Whitney *U* tests revealed statistically significant differences in ratings for scent, taste, and aftertaste, with moderate to large effect sizes indicating moderate to large carryover effects. No significant differences were observed for overall comfort, overall procedure, and oral irritation severity, indicating no carryover effects for these variables. For binary variables, chi‐square tests revealed no significant group differences for gagging (*χ* ^2^ = 0.502, *p* = 0.479) or oral irritation experience (*χ* ^2^ = 0.254, *p* = 0.615), suggesting no carryover effects on these variables (Table 4).
5. Preference for flavored or non‐flavored PSP sleeve:

**Table 2:** Within‐group median (IQR) comparisons and effect sizes for Group A (flavored first) and Group B (non‐flavored first).

Variable	Median (IQR) flavored	Median (IQR) non‐flavored	Effect size (*r*)	*p*‐value
**Group A**				
Overall comfort	2 (2–3)	3 (2–3)	0.31	0.073
Overall procedure	3 (3–4)	3 (3–3)	0.598	**0.002**
Scent	3 (3–4)	3 (3–3)	0.824	**< 0.001**
Taste	4 (3–4)	3 (3–3)	0.688	**0.002**
Aftertaste	4 (3–4)	3 (3–3)	0.591	**< 0.001**
Feel of PSP	3 (2–3)	3 (2–3)	0.0	1.000
Oral irritation severity	0	0	0.45	0.234
**Group B**				
Overall comfort	3 (2–4)	3 (2–4)	0.16	0.443
Overall procedure	3 (3–3)	3.5 (3–4)	0.008	0.976
Scent	3 (3–4)	3 (3–3)	0.886	**< 0.001**
Taste	4 (3–4)	3 (3–3)	0.874	**< 0.001**
Aftertaste	3.5 (3–4)	3 (3–3)	0.952	**< 0.001**
Feel of PSP	3 (2–3.25)	2 (2–3)	0.557	**0.018**
Oral irritation severity	0	0	0.44	0.165

*Note:* Significant *p* values are in bold. (Effect size interpretation [Cohen's *r*]: < 0.3 small; 0.3–0.5 medium effect; > 0.50 large effect).

**Table 3:** Order effects between Group A and Group B.

Variable	*Z*	*p*‐value	Effect size (*r*)
Overall comfort	−2.626	**0.009**	0.30
Overall procedure	−1.800	0.072	0.21
Scent	−4.470	**< 0.01**	0.52
Taste	−5.124	**< 0.01**	0.59
Aftertaste	−4.383	**< 0.01**	0.51
Feel of PSP	−1.092	0.275	0.13
Oral irritation severity	−0.580	0.562	0.07

*Note:* Significant *p* values are in bold. (Cohen's *r*: < 0.3 small; 0.3–0.5 medium effect; > 0.50 large effect).

**Table 4:** Carryover effects between Group A and Group B.

Variable	*Z*	*p*‐value	Effect size (*r*)
Overall comfort	−0.755	0.450	0.09
Overall procedure	−0.418	0.676	0.048
Scent	−4.332	**< 0.001**	0.500
Taste	−4.591	**< 0.001**	0.530
Aftertaste	−3.278	**0.001**	0.387
Feel of PSP	−1.745	0.081	0.201
Oral irritation	−0.489	0.625	0.06

*Note:* Significant *p* values are in bold. (Cohen's *r*: < 0.3 small; 0.3 −0.5 medium effect; > 0.50 large effect).

After completing both study phases, participants were asked which PSP sleeve they preferred. Overall, 58.7% (*n* = 44) preferred the flavored sleeve, while 41.3% (*n* = 31) preferred the non‐flavored sleeve (*p* = 0.165). However, when the preference based on treatment order was compared, a statistically significant association was found (*χ* ^2^ (1) = 11.701, *p* < 0.001). Group A (who received the flavored sleeve first) vastly preferred it (29 out of 37, 78.4%), while in Group B (those who received the non‐flavored first), 60.5% (23 out of 38) preferred the non‐flavored sleeve. This indicates that participants preferred the sleeve they experienced first (an order effect).

## 4 Discussion

This double‐blind, randomized crossover study evaluated whether mint‐flavor enhanced PSP barrier sleeves improved participant comfort and sensory experience during bitewing radiography. Flavored PSP sleeves reliably improved taste, aftertaste, and scent; these improvements were supported by higher median scores and wider IQRs, indicating both greater satisfaction and consistency of the sensory experience. By contrast, effects on procedural comfort and overall ratings were modest and contingent on exposure order.

A pronounced sequence (first exposure) effect was observed, with participants tending to favor the condition they encountered first. This suggests a potential primacy effect, (Anderson 1965) where initial impressions shape subsequent evaluations. Improvements in overall procedure ratings were most evident when flavoring was experienced first, with those in the non‐flavored group forming less favorable global impressions that persisted even after later exposure to flavoring. Taken together, our findings suggest that flavoring improves the sensory profile of PSP sleeves, while global comfort judgments are partly shaped by initial exposure.

These results help clarify the role of chemosensory cues in intraoral imaging. Flavoring appears to enhance acceptability rather than resolve the mechanical discomfort arising from sensor bulk, soft‐tissue contact, or triggering the gag reflex. Consistent with this, gagging, oral irritation, and tactile feel did not differ meaningfully between conditions. Nonetheless, a more pleasant sensory backdrop may still be clinically useful by making the procedure more tolerable, particularly in first‐time patients, children or anxious individuals. The carryover effects were stronger in the chemosensory judgments (taste, scent, and aftertaste) but not in the non‐sensory endpoints (comfort, feel, oral irritation, and gagging). Given the brief intervention and adequate washout, the practical significance of this carryover is likely limited to lingering taste or smell memory rather than true procedural effects.

Flavored PSP sleeves are inexpensive, easy to implement, and compatible with routine clinical workflows. Our study demonstrates their use as a pragmatic adjunct to enhance the sensory acceptability of bitewing placement, particularly for patients whose cooperation may be influenced by initial impressions. The findings of this study align with prior research suggesting that sensory enhancements and modifications can improve patients’ subjective experiences during clinical procedures (Lehrner et al. 2000; Toet et al. 2010; Hattab et al. 1999; Kumar and Jessy 2020; Cermak et al. 2015). While this modification may not drastically reduce discomfort or exaggerated gagging reflexes, it could help improve patient cooperation and reduce anxiety, particularly in new dental patients, children, and individuals with special needs. Added flavoring to PSP sleeves may potentially contribute to greater comfort, reduced anxiety, and increased cooperation during intraoral radiography, all factors that could decrease the need for repeat radiographs.

To our knowledge, this is the first study to investigate the effect of flavored PSP barrier sleeves on sensory experience and procedural comfort during intraoral radiography. This underscores the novelty of our findings and highlights the need for further research to explore modifications in intraoral radiographic procedures to improve patient comfort.

### 4.1 Strengths and Limitations

A key strength of this study was the double‐blind crossover design, which maximized the reliability and validity of the findings while minimizsing bias and variability. This design is particularly suited for evaluating the effects of flavored and non‐flavored PSP barrier sleeves for several reasons. In a crossover design, each participant serves as their own control. This approach reduces inter‐observer variability (such as differences in oral sensitivity, gagging tendencies, or comfort thresholds), thereby enhancing the precision of comparisons between the treatment (flavored) and the control (non‐flavored). The blinding setup ensured that neither the participants nor the investigators were aware of the allocation of treatment or control. Although the study was designed as a double‐blind trial, the distinct sensory difference between the treatment and control (the taste of the barrier sleeve) may have led participants to infer their allocation during phase 2 of the crossover. This potential unblinding could have influenced subjective responses, particularly those related to taste and overall comfort. In addition, the use of self‐reported data introduces subjectivity, and the study population, comprising dental students, limits the generalizability of the findings.

Future studies should include broader and more diverse patient populations, including children and individuals with known exaggerated gag reflexes. Further research should also consider other flavors, long‐term acceptability, cost‐effectiveness, and potential placebo effects of sensory modifications of PSP barrier sleeves.

## 5 Conclusion

Flavoring PSP barrier sleeves improved sensory experiences during bitewing radiography but had a limited influence on procedural discomfort. These findings suggest that while sensory enhancements can improve the patient experience, they may not be sufficient on their own to address procedural discomfort or exaggerated gag reflexes. Nonetheless, they represent a low‐cost, practical approach to enhance patient cooperation and overall experience in dental radiographic procedures.

## Author Contributions

**Shwetha Hegde:** conceptualization, design, methodology, project administration, investigation and formal analysis, visualization, writing the original draft and revising the manuscript. **Wendy Currie:** conceptualization, design, methodology, feedback on the concept, writing (review and editing) and visualization. **Shanika Nanayakkara:** formal analysis, visualization and writing (review and editing). **Scott Cameron Dickie:** project administration, investigations, methodology, review and editing of the manuscript. **Thirimadura Ruvin Mendis:** investigations, methodology, review and editing of the manuscript. **Mathew Yu:** investigations, methodology, review and editing of the manuscript. **Amelita Simpson:** Review and editing of the manuscript.

## Conflicts of Interest

The authors declare no conflicts of interest.

## Supporting information

File 1: Consent form.

File 2: Questionnaire 1.

File 3: Questionnaire 2.

SuppTables_all.

## Data Availability

The datasets generated and/or analyzed during the current study are available from the corresponding author on reasonable request.
