# Supplementary material for: An Evaluation of Flavored Photostimulable Phosphor (PSP) Barrier in Bitewing Radiography: A Randomized Crossover Study
Source: Clin Exp Dent Res. 2026 Mar 26;12(2):e70329. doi: 10.1002/cre2.70329 (PMC13140404; doi:10.1002/cre2.70329)
Supplement: Supplementary file 1 — File 1: Consent form. [file CRE2-12-e70329-s004.pdf]

Participant Consent Form

PARTICIPANT CONSENT FORM
Study title: Impact of using added flavouring to photo stimulable phosphor (PSP) barrier packets on patient experience during intraoral radiography: A comparative study

[Participant Information Statement]

Researcher's contact details
Flavoured film packets study 2022
<94c19306.groups.sydney.edu.au@au.teams.ms>

Participant name and Cohort (whether enrolled in DMD or BOH program)

[ ]

I agree to take part in this research study

- ○ Yes
- ○ No

In giving my consent, I confirm that:

	Yes	Nc
The details of my involvement have been explained to me, and I have been provided with a written Participant Information Statement to keep	○	○
I understand the purpose of the study is to investigate the effect of flavouring on the participant experience during intraoral radiography.	○	○
I acknowledge that the risks and benefits of participating in this study have been explained to me to my satisfaction	○	○
I understand that in this study I will be required to attend dental clinics at Westmead Centre of Oral Health. I understand that a PSP film will be placed in my mouth for a molar radiograph and removed and that I will not be exposed to x-rays during the procedure	○	○
I understand that my deidentified information may be used in future research to guide study design.	○	○
I understand that being in this study is completely voluntary	○	○
I am assured that my decision to participate will not have any impact on my relationship with the research team or the University of Sydney.	○	○
I understand that I am free to withdraw from this study and that I can choose to withdraw any information I have already provided (unless the data has already been de-identified or published).	○	○
I have been informed that the confidentiality of the information I provide will be protected and will only be used for purposes that I have agreed to. I understand that information about me will only be told to others with my permission, except as required by law.	○	○
I understand that the results of this study may be published and that publications will not contain my name or any identifiable information about me.	○	○

I confirm the following:

- ☐ I consent to be contacted for future studies
- ☐ I consent to my data being used in future research
- ☐ I would like feedback on the overall results of this study

If you answered yes to the previous question, please provide your preferred contact details (email/telephone/postal address):

I understand that after I sign and return this consent form it will be retained by the researcher and that I may request a copy at any time.

- ◯ Yes
- ◯ No

Participant's name and date

Powered by Qualtrics

Questions before the study starts

Participant number:

____

Questions after the second arm of the study

How would you evaluate the overall comfort of this procedure?

- ○ Very comfortable
- ○ Comfortable
- ○ Neither comfortable nor uncomfortable
- ○ Somewhat Uncomfortable
- ○ Very uncomfortable

Please indicate why the overall procedure was uncomfortable for you. You may choose more than one.

- ☐ The procedure made me gag
- ☐ The plate tasted unpleasant
- ☐ The plate smelled unpleasant
- ☐ The plate size was too big
- ☐ The plate left a bad aftertaste
- ☐ The plate was in my mouth for an uncomfortable amount of time
- ☐ Before the procedure began, I was feeling anxious, worried, upset or tense about any aspect of the procedure

☐ During the procedure, I was feeling anxious, worried, upset or tense about any aspect of the procedure

☐ [ ] Other, please specify

Please answer the following questions.

	Relaxed	A little uneasy	Tense	Anxious	So anxious that I almost felt physically sick
While you were waiting to take part in this procedure, how did you feel?	○	○	○	○	○
During the length of the procedure, how did you feel?	○	○	○	○	○

Did you experience gagging during this procedure?

○ Yes

○ No

Please rate how strong you would say your gag reflex was.

Not strong at all				Very strong
○	○	○	○	○

Did you experience any oral irritation ?

○ Yes

○ No

Please indicate the level of oral irritation you experienced with the PSP plate

- ◯ Very High
- ◯ High
- ◯ Somewhat high
- ◯ Low, noticeable irritation
- ◯ Very low, did not bother me

Please indicate your experience with each of the following:

	Very Unpleasant	Unpleasant	Neither Pleasant or Unpleasant	Somewhat Pleasant
Overall Procedure	◯	◯	◯	◯
Scent of the PSP	◯	◯	◯	◯
Taste of the PSP	◯	◯	◯	◯
Aftertaste of the PSP	◯	◯	◯	◯
Feel of the PSP	◯	◯	◯	◯

Comparator questions

Which plate did you prefer overall?

- ◯ Plate A
- ◯ Plate B

Which of these factors, if any, contributed to your decision?

- ◯ Feel of the plate
- ◯ Scent of the plate
- ◯ Taste of the plate
- ◯ Aftertaste of the plate
- ◯ Comfort of the overall procedure
- ◯ [ ] Other, please specify

Powered by Qualtrics

Block 1

Participant number:

Are you currently feeling unwell or have any of the following symptoms:
- Fever
- Dry cough
- Sore throat
- Lethargy

- ◯ Yes
- ◯ No

Have you been overseas in the last 14 days or do you believe you have been in contact with anyone who may have COVID-19 in that time?

- ◯ Yes
- ◯ No

Are you a smoker?

- ◯ Yes
- ◯ No

Are you currently recovering from oral surgery?

- Yes
- No

Are you currently pregnant or likely to be pregnant?

- Yes
- No

Do you have a known allergy to breath freshener sprays or any of the following ingredients:
Ethanol, glycerol, Peg-40 castor oil, sodium saccharin, menthol, acetyl pyridinium chloride, brilliant blue FCF Ci42090 food colouring.

- Yes
- No

Questions before the study starts

Have had intraoral radiographs taken before?

- Yes
- No

How do you rate your previous experience of having an intraoral radiograph taken?

- Extremely good
- Somewhat good
- Neither good nor bad
- Somewhat bad

- ◯ Extremely bad

Please indicate why your previous experience with intraoral radiographs was bad. You may choose more than one.

- ☐ I was feeling worried, tense or upset about the idea of having an intraoral radiograph taken
- ☐ The plate was in my mouth for an uncomfortable amount of time
- ☐ The plate size was too big
- ☐ The plate tasted or smelled unpleasant
- ☐ The plate left a bad aftertaste
- ☐ The procedure caused me to gag
- ☐ ________ Other, please specify

Do you have a gag reflex?

- ◯ Yes
- ◯ No

How strong would you say your gag reflex is?

Not strong at all				Very Strong
◯	◯	◯	◯	◯

Have you ever had a negative experience with gagging?

- ◯ Yes
- ◯ No

Have you ever gagged at the dentist before?

- ◯ Yes
- ◯ No

Please select any of the following procedures that have caused you to gag:

- ☐ Impression taking
- ☐ Application of orthodontic appliances like braces or expanders
- ☐ Root canal treatment
- ☐ Cavity filling
- ☐ Intraoral Radiograph
- ☐ Teeth cleaning
- ☐ ______ Other, please specify:

Please rank the following flavours based on your personal preference.

Strawberry

Mint

Bubblegum

Chocolate

Vanilla

Unflavoured

Questions after the first arm of the study

How would you evaluate the overall comfort of this procedure?

- ○ Very comfortable
- ○ Comfortable
- ○ Neither comfortable nor uncomfortable
- ○ Somewhat Uncomfortable
- ○ Very uncomfortable

Please indicate why the overall procedure was uncomfortable for you. You may choose more than one.

- ☐ The procedure made me gag
- ☐ The plate tasted unpleasant
- ☐ The plate smelled unpleasant
- ☐ The plate size was too big
- ☐ The plate left a bad aftertaste
- ☐ The plate was in my mouth for an uncomfortable amount of time
- ☐ Before the procedure began, I was feeling anxious, worried, upset or tense about any aspect of the procedure
- ☐ During the procedure, I was feeling anxious, worried, upset or tense about any aspect of the procedure
- ☐ ______ Other, please specify

Please answer the following questions.

	Relaxed	A little uneasy	Tense	Anxious	So anxious that I almost felt physically sick
While you were waiting to take part in this procedure, how did you feel?	○	○	○	○	○
During the length of the procedure, how did you feel?	○	○	○	○	○

Did you experience gagging during this procedure?

- ○ Yes
- ○ No

Please rate how strong you would say your gag reflex was.

Not strong at all				Very strong
◯	◯	◯	◯	◯

Did you experience any oral irritation ?

◯ Yes

◯ No

Please indicate the level of oral irritation you experienced with the PSP plate

◯ Very High

◯ High

◯ Somewhat high

◯ Low, noticeable irritation

◯ Very low, did not bother me

Please indicate your experience with each of the following:

	Very Unpleasant	Unpleasant	Neither Pleasant or Unpleasant	Somewhat Pleasant
Overall Procedure	◯	◯	◯	◯
Scent of the PSP	◯	◯	◯	◯
Taste of the PSP	◯	◯	◯	◯
Aftertaste of the PSP	◯	◯	◯	◯
Feel of the PSP	◯	◯	◯	◯

Powered by Qualtrics
