# Supplementary material for: An Evaluation of Flavored Photostimulable Phosphor (PSP) Barrier in Bitewing Radiography: A Randomized Crossover Study
Source: Clin Exp Dent Res. 2026 Mar 26;12(2):e70329. doi: 10.1002/cre2.70329 (PMC13140404; doi:10.1002/cre2.70329)
Supplement: Supplementary file 2 — File 2: Questionnaire 1. [file CRE2-12-e70329-s001.pdf]

## Block 1

Participant number:

Are you currently feeling unwell or have any of the following symptoms:
- Fever
- Dry cough
- Sore throat
- Lethargy

- ◯ Yes
- ◯ No

Have you been overseas in the last 14 days or do you believe you have been in contact with anyone who may have COVID-19 in that time?

- ◯ Yes
- ◯ No

Are you a smoker?

- ◯ Yes
- ◯ No

Are you currently recovering from oral surgery?

- Yes
- No

Are you currently pregnant or likely to be pregnant?

- Yes
- No

Do you have a known allergy to breath freshener sprays or any of the following ingredients:
Ethanol, glycerol, Peg-40 castor oil, sodium saccharin, menthol, acetyl pyridinium chloride, brilliant blue FCF Ci42090 food colouring.

- Yes
- No

**Questions before the study starts**

Have had intraoral radiographs taken before?

- Yes
- No

How do you rate your previous experience of having an intraoral radiograph taken?

- Extremely good
- Somewhat good
- Neither good nor bad
- Somewhat bad

◯ Extremely bad

Please indicate why your previous experience with intraoral radiographs was bad. You may choose more than one.

- ☐ I was feeling worried, tense or upset about the idea of having an intraoral radiograph taken
- ☐ The plate was in my mouth for an uncomfortable amount of time
- ☐ The plate size was too big
- ☐ The plate tasted or smelled unpleasant
- ☐ The plate left a bad aftertaste
- ☐ The procedure caused me to gag
- ☐ ________ Other, please specify

Do you have a gag reflex?

◯ Yes

◯ No

How strong would you say your gag reflex is?

| Not strong at all | | | | Very Strong |
|---|---|---|---|---|
| ◯ | ◯ | ◯ | ◯ | ◯ |

Have you ever had a negative experience with gagging?

◯ Yes

◯ No

Have you ever gagged at the dentist before?

◯ Yes

◯ No

Please select any of the following procedures that have caused you to gag:

- [ ] Impression taking
- [ ] Application of orthodontic appliances like braces or expanders
- [ ] Root canal treatment
- [ ] Cavity filling
- [ ] Intraoral Radiograph
- [ ] Teeth cleaning
- [ ] ________ Other, please specify:

Please rank the following flavours based on your personal preference.

Strawberry

Mint

Bubblegum

Chocolate

Vanilla

Unflavoured

## Questions after the first arm of the study

How would you evaluate the overall comfort of this procedure?

- ○ Very comfortable
- ○ Comfortable
- ○ Neither comfortable nor uncomfortable
- ○ Somewhat Uncomfortable
- ○ Very uncomfortable

Please indicate why the overall procedure was uncomfortable for you. You may choose more than one.

- ☐ The procedure made me gag
- ☐ The plate tasted unpleasant
- ☐ The plate smelled unpleasant
- ☐ The plate size was too big
- ☐ The plate left a bad aftertaste
- ☐ The plate was in my mouth for an uncomfortable amount of time
- ☐ Before the procedure began, I was feeling anxious, worried, upset or tense about any aspect of the procedure
- ☐ During the procedure, I was feeling anxious, worried, upset or tense about any aspect of the procedure
- ☐ ____________ Other, please specify

Please answer the following questions.

| | Relaxed | A little uneasy | Tense | Anxious | So anxious that I almost felt physically sick |
|---|---|---|---|---|---|
| While you were waiting to take part in this procedure, how did you feel? | ○ | ○ | ○ | ○ | ○ |
| During the length of the procedure, how did you feel? | ○ | ○ | ○ | ○ | ○ |

Did you experience gagging during this procedure?

- ○ Yes
- ○ No

Please rate how strong you would say your gag reflex was.

| Not strong at all | | | | Very strong |
|---|---|---|---|---|
| ◯ | ◯ | ◯ | ◯ | ◯ |

Did you experience any oral irritation ?

◯ Yes

◯ No

Please indicate the level of oral irritation you experienced with the PSP plate

◯ Very High

◯ High

◯ Somewhat high

◯ Low, noticeable irritation

◯ Very low, did not bother me

Please indicate your experience with each of the following:

| | Very Unpleasant | Unpleasant | Neither Pleasant or Unpleasant | Somewhat Pleasant |
|---|---|---|---|---|
| Overall Procedure | ◯ | ◯ | ◯ | ◯ |
| Scent of the PSP | ◯ | ◯ | ◯ | ◯ |
| Taste of the PSP | ◯ | ◯ | ◯ | ◯ |
| Aftertaste of the PSP | ◯ | ◯ | ◯ | ◯ |
| Feel of the PSP | ◯ | ◯ | ◯ | ◯ |

Powered by Qualtrics
