# Supplementary material for: An Evaluation of Flavored Photostimulable Phosphor (PSP) Barrier in Bitewing Radiography: A Randomized Crossover Study
Source: Clin Exp Dent Res. 2026 Mar 26;12(2):e70329. doi: 10.1002/cre2.70329 (PMC13140404; doi:10.1002/cre2.70329)
Supplement: Supplementary file 3 — File 3: Questionnaire 2. [file CRE2-12-e70329-s003.pdf]

Questions before the study starts

Participant number:

Questions after the second arm of the study

How would you evaluate the overall comfort of this procedure?

- ◯ Very comfortable
- ◯ Comfortable
- ◯ Neither comfortable nor uncomfortable
- ◯ Somewhat Uncomfortable
- ◯ Very uncomfortable

Please indicate why the overall procedure was uncomfortable for you. You may choose more than one.

- ☐ The procedure made me gag
- ☐ The plate tasted unpleasant
- ☐ The plate smelled unpleasant
- ☐ The plate size was too big
- ☐ The plate left a bad aftertaste
- ☐ The plate was in my mouth for an uncomfortable amount of time
- ☐ Before the procedure began, I was feeling anxious, worried, upset or tense about any aspect of the procedure

- ☐ During the procedure, I was feeling anxious, worried, upset or tense about any aspect of the procedure
- ☐ ________ Other, please specify

Please answer the following questions.

	Relaxed	A little uneasy	Tense	Anxious	So anxious that I almost felt physically sick
While you were waiting to take part in this procedure, how did you feel?	○	○	○	○	○
During the length of the procedure, how did you feel?	○	○	○	○	○

Did you experience gagging during this procedure?

- ○ Yes
- ○ No

Please rate how strong you would say your gag reflex was.

Not strong at all				Very strong
○	○	○	○	○

Did you experience any oral irritation ?

- ○ Yes
- ○ No

Please indicate the level of oral irritation you experienced with the PSP plate

- ◯ Very High
- ◯ High
- ◯ Somewhat high
- ◯ Low, noticeable irritation
- ◯ Very low, did not bother me

Please indicate your experience with each of the following:

	Very Unpleasant	Unpleasant	Neither Pleasant or Unpleasant	Somewhat Pleasant
Overall Procedure	◯	◯	◯	◯
Scent of the PSP	◯	◯	◯	◯
Taste of the PSP	◯	◯	◯	◯
Aftertaste of the PSP	◯	◯	◯	◯
Feel of the PSP	◯	◯	◯	◯

Comparator questions

Which plate did you prefer overall?

- ◯ Plate A
- ◯ Plate B

Which of these factors, if any, contributed to your decision?

- ◯ Feel of the plate
- ◯ Scent of the plate
- ◯ Taste of the plate
- ◯ Aftertaste of the plate
- ◯ Comfort of the overall procedure
- ◯ [ ] Other, please specify

Powered by Qualtrics
