# Supplementary material for: An Evaluation of Flavored Photostimulable Phosphor (PSP) Barrier in Bitewing Radiography: A Randomized Crossover Study
Source: Clin Exp Dent Res. 2026 Mar 26;12(2):e70329. doi: 10.1002/cre2.70329 (PMC13140404; doi:10.1002/cre2.70329)
Supplement: Supplementary file 4 — SuppTables_all. [file CRE2-12-e70329-s002.docx]

**Supplementary tables:**

| **Flavour** | **Mean rank** |
| --- | --- |
| Mint | 2.49 |
| Strawberry | 2.78 |
| Vanilla | 3.64 |
| Chocolate | 3.73 |
| Bubble gum | 3.75 |
| Unflavoured | 4.62 |

Supplementary Table S1: Mean ranks for preference for different PSP flavourings.

| **Variable** | **Median (IQR) Flavoured** | **Median (IQR) Non-flavoured** | **p-value** | **Effect size** |
| --- | --- | --- | --- | --- |
| Overall comfort | 4.0 (2-4) | 3.0 (2-4) | **0.045** | 0.231 |
| Overall procedure | 3.0 (3-4) | 3.0 (3-3) | **0.043** | 0.233 |
| Scent | 3.0 (3-4) | 3.0 (3-3) | **<0.001** | 0.641 |
| Aftertaste | 4.0 (3-4) | 3.0 (3-3) | **<0.001** | 0.576 |
| Taste | 4.0 (3-4) | 3.0 (3-3) | **<0.001** | 0.649 |
| Feel | 3.0 (2-3) | 3.0 (2-3) | **0.043** | 0.234 |
| Oral irritation severity | 0 | 0 | 0.905 | 0.029 |

Supplementary Table S2: Comparison of median scores of participant responses to flavoured and non-flavoured PSP barrier sleeves across different variables, interquartile range, p values and effect size for the variables rated on an ordinal scale; Significant p values are in bold. (Cohen's r: <0.3 small; 0.3 -0.5 medium effect; >0.50 large effect).
